# Colorectal Cancer Growth Retardation through Induction of Apoptosis, Using an Optimized Synergistic Cocktail of Axitinib, Erlotinib, and Dasatinib

**DOI:** 10.3390/cancers11121878

**Published:** 2019-11-27

**Authors:** Robert H. Berndsen, Nathalie Swier, Judy R. van Beijnum, Patrycja Nowak-Sliwinska

**Affiliations:** 1 Molecular Pharmacology Group, Institute of Pharmaceutical Sciences of Western Switzerland, University of Geneva, Rue Michel-Servet 1, 1211 Geneva, Switzerland; bobberndsen@hotmail.com; 2 Angiogenesis Laboratory, Department of Medical Oncology, Cancer Center Amsterdam, Amsterdam UMC-Location VUmc, VU University Amsterdam, De Boelelaan 1117, 1081 HV Amsterdam, The Netherlands; nathalieswier@hotmail.com (N.S.); judy.vanbeijnum@gmail.com (J.R.v.B.); 3 Translational Research Center in Oncohaematology, Rue Michel-Servet 1, 1211 Geneva, Switzerland

**Keywords:** drug combination, colorectal carcinoma, phosphokinase analysis, chicken chorioallantoic membrane

## Abstract

Patients with advanced colorectal cancer (CRC) still depend on chemotherapy regimens that are associated with significant limitations, including resistance and toxicity. The contribution of tyrosine kinase inhibitors (TKIs) to the prolongation of survival in these patients is limited, hampering clinical implementation. It is suggested that an optimal combination of appropriate TKIs can outperform treatment strategies that contain chemotherapy. We have previously identified a strongly synergistic drug combination (SDC), consisting of axitinib, erlotinib, and dasatinib that is active in renal cell carcinoma cells. In this study, we investigated the activity of this SDC in different CRC cell lines (SW620, HT29, and DLD-1) in more detail. SDC treatment significantly and synergistically decreased cell metabolic activity and induced apoptosis. The translation of the in-vitro-based results to in vivo conditions revealed significant CRC tumor growth inhibition, as evaluated in the chicken chorioallantoic membrane (CAM) model. Phosphoproteomics analysis of the tested cell lines revealed expression profiles that explained the observed activity. In conclusion, we demonstrate promising activity of an optimized mixture of axitinib, erlotinib, and dasatinib in CRC cells, and suggest further translational development of this drug mixture.

## 1. Introduction

Patients with primary or secondary metastatic colorectal cancer (mCRC) still depend on chemotherapy regimens, with or without surgical intervention [1]. This therapy includes irinotecan, oxaliplatin, fluorouracil (5-FU), leucovorin or capecitabine, mostly administered in combination regimens (e.g., FOLFIRI/FOLFOX or CAPIRI/CAPOX). Limitations to the use of these drugs include acquired or intrinsic resistance [2], tumor and patient heterogeneity [3], and toxicities [4]. As colorectal cancer is one of the most prevalent cancer types and is associated with a high number of cancer-related deaths, numerous studies have been performed over the last decades, aimed at developing therapies that improve patient survival and decrease disease morbidity.

In patients with advanced CRC, a combined treatment of bevacizumab (targeting VEGF) or cetuximab (targeting EGFR), and chemotherapy regimens led to a modest increase of 2–6 months in overall survival [5]. Another example is regorafenib (targeting VEGFR-2 and TIE2), which was found to improve overall survival, as monotherapy in patients have shown disease progression even after prior standard therapies [6]. The clinical use of these drugs individually, however, is rather limited since the survival benefit is seen only in small groups of patients, and therapy is associated with significant toxicities and high costs [5].

An alternative treatment strategy that might be successful is the use of combinations of drugs that are designed to be specific for a certain tumor type or are repurposed from another indication but intercalate a specific signaling pathway [7]. When targeted drugs are combined, multiple non-overlapping molecular pathways can be inhibited, thus, tackling the robustness of cancer signaling pathways. This might lead to an increased efficacy and delayed onset of drug resistance [8,9]. Moreover, identifying synergistic interactions has the potential to maximize efficacy while minimizing dose levels and, therefore, decreasing the probability of significant toxicities [9]. However, the search for combinations of tyrosine kinase inhibitors (TKIs) [10], checkpoint inhibitors [11], chemotherapeutic agents [12], epigenetic drugs [13], and microRNAs [14] seems to have just begun. Recently, a phase III clinical trial in patients with BRAF-mutated mCRC showed increased overall survival when treated with a combination of the targeted drugs encorafenib (targeting BRAF), binimetinib (targeting MEK), and cetuximab (targeting EGFR), as compared to the standard care of chemotherapy without leading to increased toxicities [15]. This indicates the potential of combinations of TKIs in patients with advanced CRC.

To identify optimal drug combinations, a number of initiatives made use of artificial intelligence [16,17]. Likewise, we have previously developed and validated a method, called the streamlined-Feedback System Control (s-FSC) technique, which rapidly identifies synergistic drug combinations, making use of simple in vitro bioassays [18,19]. The major advantages of this technique are that (i) it does not require mechanistic input, (ii) it can identify non-obvious drug combinations, and (iii) it samples only a small portion of possible combinations to be tested experimentally [20,21]. Rather than tediously determining the best concentration for each drug individually, the s-FSC workflow together with a stepwise regression coefficient analysis can efficiently extract this information with a surprisingly limited set of experimental tests. Hence, the power of s-FSC is that it provides an efficient framework to identify combinations of multiple drugs. This constitutes a quantum leap, compared to the more classical combinations of two drugs at a time, as mentioned before [22,23].

Using this method, employing a simple in vitro cell metabolic activity assay, we identified a synergistic drug combination (SDC) containing axitinib (targeting VEGFR, PDGFR, FGFR1, and c-KIT), erlotinib (targeting EGFR), and dasatinib (targeting bcr-abl and Src), which was optimized for a specific population of clear cell renal cell carcinoma (RCC) cells, 786-O [20]. Regression analysis identified synergies between axitinib and erlotinib (* *p* < 0.05) [20]. When we tested the efficacy of the SDC in other cell lines as part of a cross-validation, we noticed its strong efficacy in various CRC cells.

In this study, we validated the efficacy of the above-mentioned drug combination in CRC cells and observed strong synergistic interactions at various dose levels, as well as an efficient induction of apoptosis. To understand the resemblance in observed efficacy between the RCC cell line 786-O (used in the optimization study of the SDC) and the CRC cell lines investigated in this study, we performed a comparative analysis of phosphokinase expression profiles. This analysis showed that the key kinases targeted by the drug combination have a comparable activation profile in the RCC cell line 786-O and CRC cell lines. Moreover, we established and compared the growth of three CRC cell lines on the chicken chorioallantoic membrane (CAM) model [24,25,26]. We successfully translated the in vitro-based SDC activity in CRC tumors xenografted on the CAM and observed a significant tumor growth inhibition.

## 2. Results

### 2.1. Axitinib, Erlotinib, and Dasatinib Combination Synergistically Inhibits Cancer Cell Metabolic Activity and Induces Apoptosis

**Figure 1:**
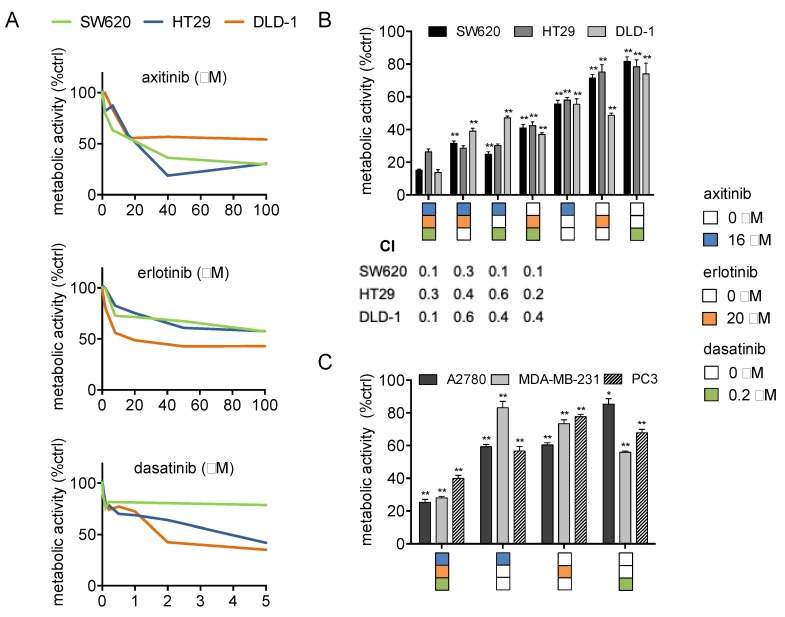
Synergistic drug combination (SDC) of axitinib, erlotinib, and dasatinib synergistically inhibits cancer cell metabolic activity. (**A**) Dose response curves of axitinib, erlotinib, and dasatinib in the CRC (SW620, HT29, DLD-1) cell lines. (**B**) Metabolic activity of the SDC compared to the two-drug combinations. CI indicates the Combination Index that was assessed using the Chou–Talalay method [27]. CI values are showed below the graph. CI < 0.9 indicates synergism, CI = 0.9 indicates an additive effect, and CI > 1 indicates antagonism. Significance (* *p* < 0.05, ** *p* < 0.01) for two-drug combinations and monotherapies is indicated as compared to the SDC. (**C**) Metabolic activity of the SDC in ovarian carcinoma (A2780), breast cancer (MDA-MB-231), and prostate cancer (PC3) cells. Significance (* *p* < 0.05, ** *p* < 0.01) is indicated as compared to control. (**A**,**B**) Color coded drug doses include axitinib 16 μM, erlotinib 20 μM, and dasatinib 0.2 μM and are based on our previous study [20]. Metabolic activity was assessed after 72 h of drug administration by the CellTiter-Glo^®^ luminescence assay and are represented as a percentage of the control. Cells treated with 0.1% DMSO were used as a control (ctrl). Error bars indicate SEM.

In previous research, using the s-FSC method, we identified a synergistic drug combination (SDC) consisting of axitinib (16 μM), erlotinib (20 μM), and dasatinib (0.2 μM) [20]. In this study, the activity of axitinib, erlotinib, and dasatinib was investigated in colorectal cancer cells (SW620, HT29, DLD-1; refer to Figure 1A for monotherapy dose response curves), at various dose levels and ratios in combination and in a variety of other cancer cell types (A2780, ovarian carcinoma; PC3, prostate cancer and MDA-MB-231, breast cancer). Exposure of CRC cells to 2- and 3-drug combinations of axitinib, erlotinib, and dasatinib significantly and synergistically inhibited the metabolic activity in SW620, HT29, and DLD-1 cells (Figure 1B; Combination Index (CI) 0.1–0.6). Notably, the SDC outperformed 2-drug combinations (similar doses as used in the SDC). Next, we evaluated to what extent dose reductions and variations in dose ratios affect the combination efficacy. Administration of 2- and 3-drug combinations at 50–90% lower doses still resulted in a synergistic inhibition of metabolic activity, however, this activity was less pronounced compared to SDC activity (Supplementary Figure S1). In addition, variations in dose ratios did not affect the combination efficacy to a great extent (Supplementary Figure S2) indicating flat and steady response surfaces around optimal SDC doses, similar to what we observed in previous studies [19,20]. In other tested cancer cell types, the SDC also resulted in a significant inhibition of metabolic activity (Figure 1C). Additionally, we tested the SDC in non-malignant cells, including peripheral blood mononuclear cells (PBMCs) and human dermal fibroblasts (HDFa), and observed the specificity towards CRC cells (Supplementary Table S1).

**Figure 2:**
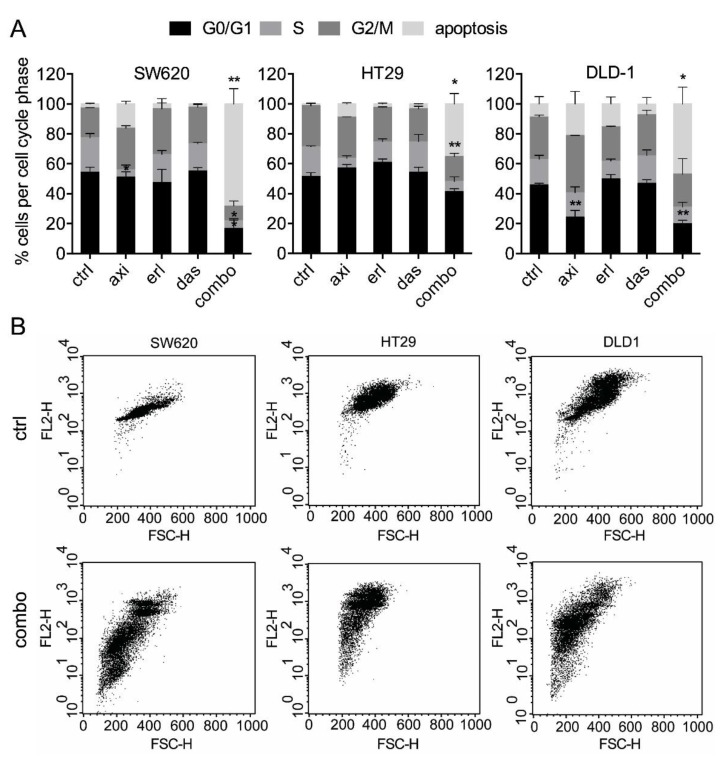
SDC axitinib, erlotinib, and dasatinib significantly induces apoptosis. (**A**) DNA distribution of cell cycle phases by quantification of propidium-iodide staining of SW620, HT29, and DLD-1 cells. Cellular DNA content in permeabilized cells is proportional to fluorescence intensity (FL2-H; y-axis), and allows for the distinction of cells containing diploid DNA (G0/G1), tetraploid DNA (G2/M), and subdiploid DNA (apoptotic fraction). (**B**) Representative FL2-H/FSC-H plots of the non-treated and SDC-treated cells (combo). All values shown are presented as percentage of the control and represent the mean of at least two experiments performed in triplicates. Cells treated with 0.1% DMSO were used as a control (ctrl). Error bars indicate SEM. Significance (* *p* < 0.05, ** *p* < 0.01) is indicated as compared to the control.

To explore the mechanism of action of the combination therapy in CRC cells, we investigated apoptosis induction levels, and cell-cycle distribution. While all single drugs induced minimal apoptosis (i.e., <15% and non-significant), the combination treatment strongly induced apoptosis (significant vs. control; Figure 2A).

**Figure 3:**
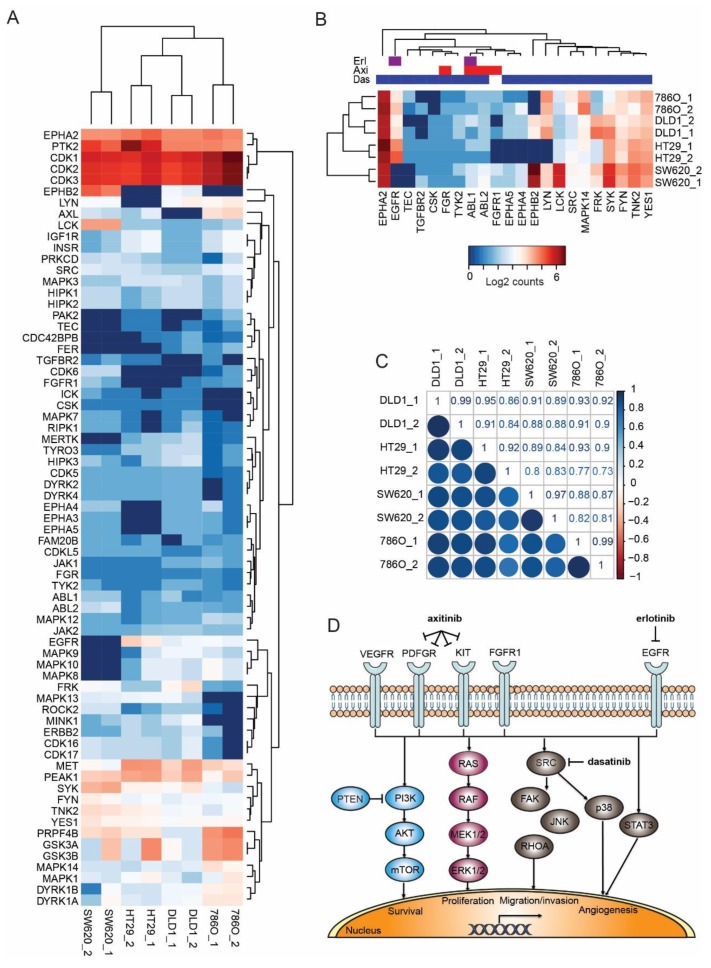
Phosphokinase comparison of colorectal cancer (CRC) cell lines SW620, HT29, and DLD-1 with the renal cell carcinoma (RCC) cell line 786-O. (**A**) Phosphoproteomics analysis of the untreated cell lines was performed on pTyr immunoprecipitated tryptic protein digest. All detected phosphorylated protein kinases over a threshold of five summed normalized spectral counts were included. The heatmap shows log2 expression of counts and indicates comparable expression profiles among the CRC line and the RCC reference line 786-O. (**B**) Phosphorylated protein kinase drug targets of the combination that was used was similar as in (**A**). Note the absence of EGFR (erlotinib target) in SW620 and the absence of FGFR1 (axitinib target) in HT29. (**C**) Correlations of phosphorylated protein kinase expression in the 4 cell lines. (**D**) Schematic overview of drug targets and their downstream signaling pathways.

To further gain insight into the responses of CRC cell lines to axitinib, erlotinib, and dasatinib combinations, we performed a comparative phosphoproteomics analysis of the three CRC cell lines and the RCC cell line 786-O. Phosphoproteomics data for these cell lines were available from ongoing studies. The analysis showed that the relative expression of most protein kinases was comparable (Figure 3A). Next, we zoomed in on the phosphokinase expression of axitinib, erlotinib, and dasatinib drug targets (Figure 3B). Drug targets were retrieved from a data repository on drug-protein interactions (www.proteomicsdb.org). Most of the kinase targets were highly expressed in all three cell lines, and most were expressed at similar levels. However, zooming in on the selected targets revealed some striking differences. Importantly, SW620 was EGFR deficient and HT29 was deficient for the major axitinib target kinase FGFR1. EPHA2 activation was high in all cell lines, whereas EPHB2 activation differed highly. Dasatinib was rather a broad spectrum TKI, targeting different members of the SRC family (including FYN, LYN, YES1, and LCK). Although these proteins differ somewhat in activation status, they serve the same pathway, and might be considered redundant to some extent. The correlation plot in Figure 3C confirms the visual similarities in phosphokinase expression between the cell lines. A schematic overview of the drugs, their targets and down-stream signaling pathways is provided in Figure 3D.

Taken together, these results indicate that the mixture of axitinib, erlotinib, and dasatinib significantly and synergistically affects the CRC cells by inhibiting metabolic activity and inducing apoptosis. The global resemblance in drug target expression between these cell lines explains their comparable sensitivity for the combination of TKI. Moreover, it shows that the mere presence or absence of a drug target does not fully determine sensitivity.

### 2.2. Axitinib, Erlotinib, and Dasatinib Combination Therapy Inhibits Colorectal Cancer Growth in the CAM Model

**Figure 4:**
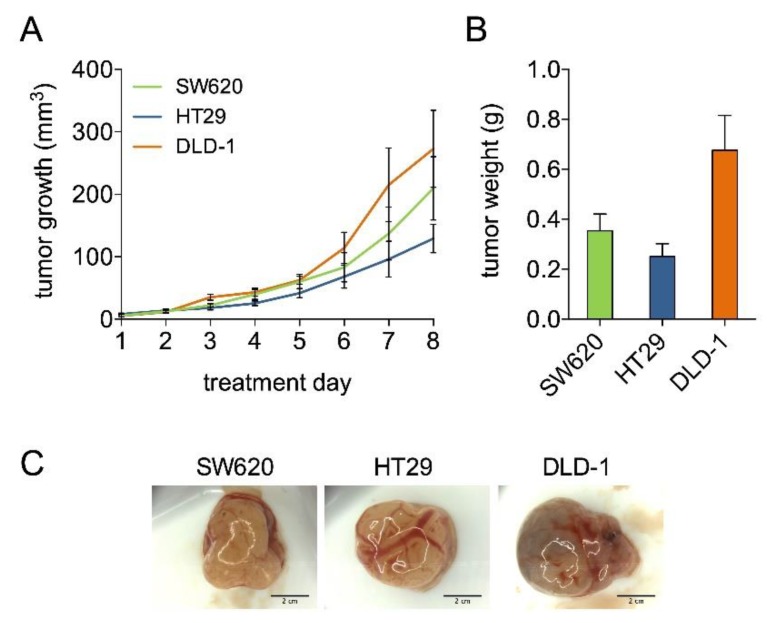
CRC tumor growth on the chicken chorioallantoic membrane (CAM). (**A**) Tumor growth curves of SW620, HT29, and DLD-1. Tumor growth is presented as tumor volume in mm^3^, with respect to days of tumor growth observation (treatment days). *N* = 3–4 per group. (**B**) Tumor weight in grams after resection on embryo development day (EDD) 18. Error bars indicate SEM. (**C**) Representative tumor images. Scale bars indicate 2 cm.

Grafting of CRC cell lines on the CAM has been described [24,28]. Here, we assessed several methods to further establish optimal tumor growth conditions of multiple cell lines (Supplementary Table S2). Suspending 1 × 10^6^ tumor cells in ice cold Geltrex™ and applying the mixture directly on the pre-treated CAM was found to be the superior method. Using this method we applied SW620, HT29, and DLD-1 cells on the CAM, resulting in various kinetics of tumor development for the three cell lines (Figure 4A,B). These tumors showed cell-line-specific morphological features such as vascularization and an erratic surface (Figure 4C).

**Figure 5:**
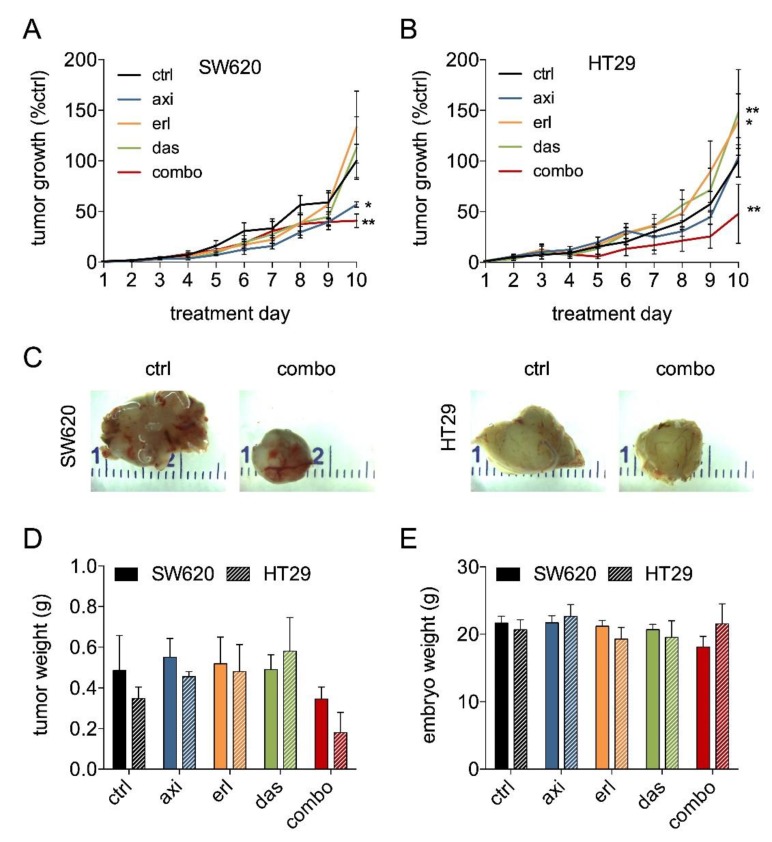
SDC axitinib, erlotinib, and dasatinib inhibits tumor growth in SW620 and HT29 tumors. Tumor growth curves of SW620 (**A**) and HT29 (**B**) treated with axitinib 72.4 μg/kg/day, erlotinib 80.6 μg/kg/day, and dasatinib 1.1 μg/kg/day. Eggs were treated with vehicle (0.1% DMSO) or drugs dissolved in sterile saline with 2 daily i.v. injections on treatment day 1 and 2 (EDD 11–12). N = 4–13 per group (**C**) Representative images of the treated and the non-treated tumors. (**D**) Tumor weight in grams on treatment day 8 (EDD 18). (**E**) Embryo weight in grams on treatment day 8 (EDD 18). Embryos were dried and weighed after tumor resection. Significance (* *p* < 0.05, ** *p* < 0.01) is indicated as compared to the control (ctrl; 0.1% DMSO treated cells) and error bars indicate SEM.

Next, we assessed the efficacy of the SDC in tumor growth inhibition in vivo. For this, SW620 and HT29 were chosen due to their distinct characteristics (Table 1) and expression of drug targets (refer to Figure 3). Following tumor grafting and randomization after visible tumors were present, treatment was performed once daily for two days on embryo development day (EDD) 11–12. In both SW620 and HT29, treatment with the SDC (axitinib 72.4 μg/kg/day, erlotinib 80.6 μg/kg/day, and dasatinib 1.1 μg/kg/day) resulted in a significant tumor growth inhibition (59.2 ± 6.7% and 52.0 ± 29.2%, respectively; Figure 5A,B). Notably, erlotinib and dasatinib monotherapy tended to stimulate tumor growth rather than inhibit it (significant only in HT29).

The presence of KRAS and BRAF mutations in these cell lines might contribute to insensitivity to erlotinib as studies showed that patients with BRAF mutations do not benefit from anti-EGFR treatment [34]. Furthermore, in SW620, administration of axitinib led to a significant tumor growth inhibition, which might be attributed to its relatively high expression of axitinib targets FGFR1 and TNIK (Figure 3B). Axitinib also targets additional angiogenic growth factor receptors (e.g., VEGFRs, PDGFRs, TIEs) that are present on tumor endothelial cells rather than on tumor cells. Ontology and pathway enrichment analysis for genes differentially expressed in HT29 and SW620 revealed that the process of angiogenesis is enriched in SW620 but not in HT29 (Supplementary Figure S3). As such, the axitinib treatment might have well-acted on both the tumor and the stromal cells in SW620. In all CAM tumor experiments, embryo weight was measured on the day of experiment termination (Figure 5E) and mortality rates were not affected by any of the treatments.

**Figure 6:**
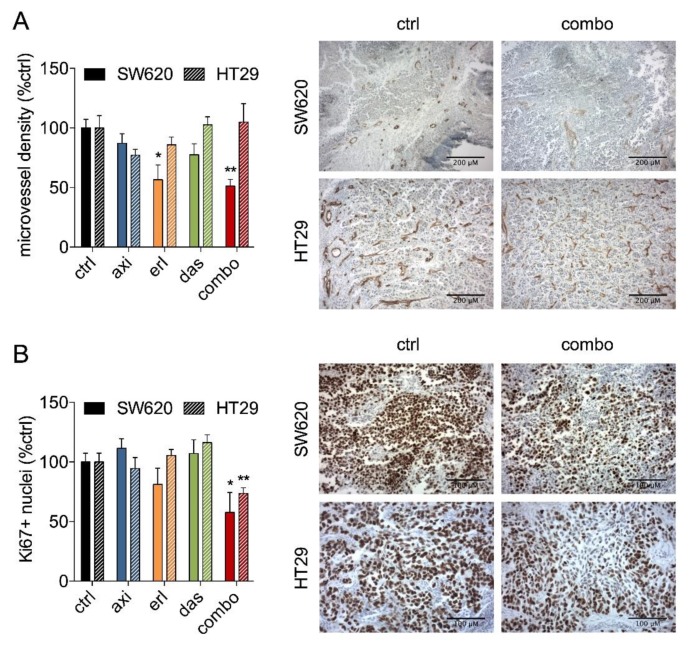
Analysis of microvessel density and the fraction of Ki67-positive cells in SW620 and HT29 CAM tumors treated with the SDC, axitinib, erlotinib, or dasatinib. (**A**) Microvessel density analysis of tumor sections stained for endothelial cell marker CD31 presented as the percentage of blood vessels per image field. Representative images of the stained tumor sections are shown. Scale bars indicate 200 µm. (**B**) Analysis of the fraction of Ki67-positive cell nuclei presented as the percentage of stained cells per image field. Representative images of the stained tumor sections are shown. Scale bars indicate 100 µm. Significance (* *p* < 0.05, ** *p* < 0.01) is indicated as compared to the control (ctrl; 0.1% DMSO-treated cells) and error bars indicate SEM.

To investigate the effect of SDC treatment on the tumor vasculature, the amount of proliferating cells and apoptosis induction, the tumor sections were stained with the endothelial cell marker CD31, the proliferation marker Ki67, and hematoxylin/eosin, respectively. Quantification of the microvessel density (MVD) revealed that only SW620 MVD was affected (Figure 6A). Furthermore, confirming the results of in vitro metabolic activity experiments (Figure 1B), quantification of Ki67 staining revealed a significant decrease in the amount of proliferating cells (Ki67 positive nuclei) in both SW620 and HT29 tumors treated with the SDC (Figure 6B). Notably, none of the single drug treatments inhibited the amount of proliferating cells in tumor sections. 

Additionally, hematoxylin/eosin (H&E) staining was performed (Supplementary Figure S4). We observed a diffuse spread of cells that showed apoptotic characteristics, such as cell shrinkage, cytoplasm condensation, and hypereosinophilic cytoplasm, in accordance to the published guidelines for recognizing apoptotic cells in H&E stained tissue [35]. In combination treated SW620 tumors, apoptotic cells appeared more widespread than in non-treated tumors, although this was not quantified.

## 3. Discussion

In previous experiments, the combination of axitinib, erlotinib, and dasatinib was optimized to target 786-O clear cell renal cell cancer carcinoma (RCC) cells, using the s-FSC technique [20]. In the present study we assessed the efficacy of this synergistic drug combination (SDC) in colorectal cancer (CRC) cells and observed strong inhibition of metabolic activity and induction of apoptosis in SW620, HT29, and DLD-1 cells. Synergistic interactions were observed at multiple dose levels and dose ratios, indicating a robust mechanism of action. Furthermore, we observed a significant tumor growth inhibition after treatment with this SDC, in an in vivo preclinical model.

Comparative phosphokinase expression analysis (Figure 3A–C) showed that, overall, the key kinases of drug targets correlate between the CRC cells and the RCC cell line 786-O, thus, explaining the observed resemblance in efficacy [20]. Since synergistic interactions due to off-target activity was described before [36], we hypothesized that here too, the combination of targeted drugs, in fact, act on multiple other protein kinases rather than only the individual-specific targets of each drug.

We used a simple method for grafting of immortalized CRC cells on the CAM that included a direct application of a suspension of 10^6^ CRC cells in Geltrex™ on the pre-treated CAM. Advantages of this technique include a fast execution of the experimental method, a need for a relatively low numbers of cells to grow tumors, and a high tumor take [24]. All three evaluated CRC cell lines grew solid tumors (Figure 4). Although the CRC cell lines have previously been reported to grow on the CAM, e.g., for SW620 [28], HT29 [37], HCT-116 [38], and SW480 [24], we have developed a technique enabling a direct comparison of the development of various CRC tumor types on the CAM. Based on our findings, we reported that the CAM model could serve as an important step, prior to the evaluation of novel treatments in more complex animal models.

The axitinib, erlotinib, and dasatinib SDC was found to inhibit tumor growth in both SW620 and HT29 CAM tumors (Figure 5). In SW620 tumors, in contrast to the HT29 tumors, administration of axitinib alone already inhibited tumor growth significantly. This result was not surprising since we already observed a stronger activity in the inhibition of metabolic activity and induction of apoptosis, as compared to erlotinib and dasatinib monotherapy (Figure 1A and Figure 2C). Moreover, although axitinib was designed as a selective VEGFR inhibitor, it has inhibitory effects on more proteins, such as FGFRs Aurora, ABL and MAPK family kinases [39,40]. Indeed, we have recently shown that targeted TKI activity is not always directly related to its designated targets [41]. Additionally, the difference in sensitivity might be explained by slightly different drug target expressions. For example, SW620 cells express FGFR1 (Figure 3B) and are sensitive to axitinib whereas HT29 cells do not express FGFR1 and lack sensitivity to axitinib.

In both SW620 and HT29, erlotinib and dasatinib were completely ineffective at inhibiting tumor growth but rather tended to stimulate tumor growth. It is unknown why this effect was seen, but it could be a compensation mechanism of the cells exposed to a treatment, as well as resistance of the cells to erlotinib, especially when exposed to low dosage [42]. Earlier studies showed that SW620 and HT29 are resistant to EGFR inhibition (cetuximab) [43], which corresponds to the lack of activity of erlotinib on tumor growth, as well as on the metabolic activity and apoptosis demonstrated here. Intriguingly, however, SW620 and HT29 were negative and positive for EGFR, respectively, suggesting different mechanisms of resistance. BRAF mutation status of HT29 might contribute to this effect.

Important to note is that when comparing the drug activity in various bioassays in vitro to tumor growth in vivo, one has to keep in mind that the tumor microenvironment consists of more than only tumor cells. Stromal cells, such as fibroblasts, endothelial cells, pericytes, and various types of immune cells that are present, are known to have a major regulatory role in the growth of tumor cells, as well as in the response of tumors to therapy and the development of drug resistance [44,45]. Moreover, varied drug sensitivities in these cell types might contribute to variable results and even observations of contradictory results, when comparing in vitro to in vivo experiments.

Blood vessel growth is a prerequisite for the growth of tumors [46,47]. As all three drugs are expected to have a direct effect on angiogenesis, we assessed the microvessel density in the treated tumors [48]. Our experiments showed that our SDC inhibited microvessel density in vivo, only in SW620, which is in line with the observed enrichment in expression of angiogenesis-related genes in SW620, as compared to HT29 (Supplementary Figure S3). In both cell lines, a decrease in the number of proliferating (Ki67 positive) cells was observed, corresponding to the inhibition of metabolic activity.

The in-vitro-optimized SDC clearly demonstrated the power of drug combination vs. individual drugs. The presented results showed a major benefit for the combination of axitinib, erlotinib, and dasatinib in the treatment of CRC and suggest the advantage of further translational development of this drug mixture.

## 4. Materials and Methods

### 4.1. Cell Lines

Colorectal cancer (CRC) cell lines (SW620, HT29 and DLD-1) were obtained from ATCC (Manassas, VA, USA) or Public Health England (Salisbury, UK), with a corresponding authentication certificate. PC3, prostate cancer; MDA-MB-231; and breast cancer cells were purchased from ATCC and A2780 ovarian carcinoma cells were purchased from Sigma (St. Louis, MO, USA). The cells were maintained in Dulbecco’s Modified Eagle’s medium (DMEM (Lonza, Basel, Switzerland), supplemented with 10% fetal bovine serum (FBS) and 1% penicillin/streptomycin (Life Technologies, Carlsbad, CA, USA). Human dermal fibroblast (HDFa) cells were purchased from Cell Applications (San Diego, CA, USA) and maintained in DMEM, supplemented as mentioned above. Human peripheral blood mononuclear cells (PBMCs) were freshly isolated as previously described [49].

### 4.2. Compounds

Axitinib and erlotinib were purchased from LC laboratories (Woburn, MA, USA). Dasatinib was purchased from Fluorochem Ltd. (Derbyshire, UK). All compounds were dissolved in sterile DMSO, stored in −80 °C, and thawed prior to each experiment. The in vitro combination experiments were performed using drug doses based on the s-FSC optimization (axitinib 6.4 and 16 μM; erlotinib 8 and 20 μM; and dasatinib 0.1 and 0.2 μM) [20].

### 4.3. Metabolic Activity Assay

For the cell metabolic activity experiments, cells were seeded in 96-well cell culture plates at a density of 3–10 × 10^3^ cells/well and were grown for 24 h. After the administration of the test compounds, the cells were allowed to grow for 72 h. Subsequently, metabolic activity was assessed with the CellTiter-Glo luminescence assay (Promega, Madison, WI, USA) that measured adenosine triphosphate (ATP) levels. Cell response to drug treatment was determined by normalizing the luminescence signal in the treated wells, as compared to the controls.

### 4.4. Apoptosis Assay

Analysis of cellular DNA content using propidium iodide was performed using flow cytometry [25]. Cells were seeded in a 24-well plate at a density of 20–40 × 10^3^ cells/well and incubated for 24 h. A medium, with or without or compounds, was applied and the cells were incubated for an additional 72 h. Cells were harvested by trypsinization and fixated in 70% ethanol for 2 h, at −20 °C. Cell pellets were then resuspended in DNA extraction buffer (90 parts 0.05 M Na_2_HPO_4_, 10 parts 0.025 M citric acid, 1 part 10% Triton-X100, pH 7.4) and incubated for 20 min at 37 °C. Propidium iodide (PI, 20 μg/mL) was added and the cells were analyzed with a FACSCalibur flow cytometer (BD Biosciences, Franklin Lakes, NJ, USA). DNA content was quantified with the CellQuest Pro software (BD Biosciences).

### 4.5. Phosphoproteome and Transcriptome Analysis

Phosphoproteomics analysis was performed, as described by van der Mijn, et al. [50]. In brief, the cells were routinely cultured in dishes, prior to lysis. Lysates were cleared by sonication, the proteins were digested by trypsin and immunoprecipitated for tyrosine phosphorylated residues. Phosphopeptides were subject to LC–MS/MS and the obtained spectra were searched against Uniprot. Phosphopeptides were assembled to phosphogenes and subsequently to phosphokinases. Normalized spectral counts were taken as a representative measure in correlation analysis, and log2 transformed for plotting in heatmaps, for a visual representation.

For the transcriptome analysis, the cell lines were grown under regular culture conditions and harvested for RNA isolation, using RNAeasy Mini Plus spin columns, in duplicates. Sample quantity and quality were checked on an Agilent biochip analyzer 2100. All samples had a RIN (RNA integrity number) of 10. Library preparation, RNA sequencing, and data processing were performed at the Institute of Genetics and Genomics of Geneva (iGE3, Geneva, Switzerland), using the SMART-Seq v4 Ultra Low Input RNA kit (Takara-Bio., Saint-Germain-en-Laye, France) for cDNA amplification, combined with the Nextera technology (Illumina, San Diego, CA, USA) for library preparation. RNA seq count data were analyzed with DEseq2 in R (version 3.5.1, available through Bioconductor.org) using RStudio (version 1.1.456, available through Bioconductor.org), using standard parameters on log2 transformed counts. Differences in transcriptome between the cell lines was further evaluated using protein interaction mapping (http://string-db.org) and ontology enrichment analysis (http://amp.pharm.mssm.edu/Enrichr/). Target kinases for the combination treatment were retrieved from a data repository on drug-kinase interactions in cell lysate (https://www.proteomicsdb.org/) [39,40], using the drug concentrations applied in this study.

### 4.6. Colorectal Cancer Tumors Grown on the Chicken Chorioallantoic Membrane (CAM)

Dutch legislation does not necessitate the acquisition of approval from institutional or licensing committee for experiments on the CAM, when terminated before hatching of the chicken embryo. Fertilized chicken eggs were incubated in a hatching incubator (humidity 65%, 37 °C). Several methods of application were evaluated to obtain an optimal tumor take.

Variables included the number of cells (0.5–5 × 10^6^ cells), the membrane matrix (Geltrex™ LDEV-Free Basement Membrane Matrix—Gibco, Carlsbad, USA versus Matrigel™—Corning, New York, USA), method of application (hanging drop versus direct application), and addition of the conditioned medium (CM) or fibroblasts. For the experiments included here, on embryo development day (EDD) 8, a mixture of 10^6^ CRC cells in the 25 μL ice cold Geltrex™ LDEV-Free Basement Membrane Matrix (Gibco, Carlsbad, CA, USA) was applied directly onto the CAM surface that was pre-treated by removal of the superficial epithelial layer. Vascularized three-dimensional tumors were visible on EDD 11 and subsequently the eggs were randomized. The compounds were freshly dissolved in 0.9% NaCl and administered on EDD 11 and 12 (referred to as treatment day 1 and 2) in 25 µL i.v. injections. The injected drug doses (axitinib 72.4 μg/kg/day, erlotinib 80.6 μg/kg/day and dasatinib 1.1 μg/kg/day) were adjusted to the normalized embryo weight at EDD 11 and 12. Control tumors were treated with vehicle (0.1% DMSO in 0.9% NaCl). The tumors were monitored daily for 10 days and the tumor size was calculated with the formula: volume = [large diameter] × [perpendicular diameter]^2^ × 0.52. On the last experiment day, embryos were sacrificed and weighed. Tumors were resected, fixed, and prepared for immunohistochemistry.

### 4.7. Immunohistochemistry

Tumor sections were stained with hematoxylin/eosin to show the morphology and the apoptotic cells, CD31 to detect blood vessels, and Ki67 to detect cell proliferation [51]. Briefly, tumors were fixed overnight in zinc fixative solution, embedded in paraffin and 5 µm sections were prepared. The sections were incubated in methanol containing 0.3% H_2_O_2_, boiled in citrate buffer (10 mM, pH 6), and blocked with 5% bovine serum albumin (BSA) in PBS. Sections were incubated with primary antibodies against CD31 (1:200; clone SZ31, Dianova, Hamburg, Germany) or Ki67 rabbit anti-human Ki67, 1:100, clone SP6; Thermo Scientific, Waltham, MA, USA). The secondary antibodies included donkey anti-rat biotinylated antibodies (1:200; Jackson, Suffolk, UK) and swine anti-rabbit biotinylated antibodies (1:200; Dako, Glostrup, Denmark). Both CD31 and Ki67 staining were followed by streptavidin-HRP (1:50; Dako, Glostrup, Denmark) and visualized by 3,3′-diaminobenzidine (DAB), resulting in a brown-colored precipitate at the antigen site. Microvessel density (MVD; number of CD31+ structures per microscopic field) and the amount of Ki67 positive cells in colorectal tumors grown on the CAM was assessed by ImageJ quantification of the representative tissue areas (10–20× objective), using the color deconvolution plugin, as previously described [37].

### 4.8. Statistics and Data Correction

The data are presented as the mean of multiple independent experiments (±SEM). In the in vitro metabolic activity assays, identification of synergistic interactions was carried out using the Chou–Talalay method [27]. In the CAM tumor growth experiments and immunohistochemistry, statistical outliers were removed from the dataset using the modified Thompson Tau test. Statistical significance was determined using the one-way or two-way ANOVA test with post-hoc Dunnett’s multiple comparison test or an unpaired *t*-test (Graphpad Prism, San Diego, CA, USA). * *p* < 0.05 and ** *p* < 0.01 were considered to be statistically significant and are indicated in comparison to the control, unless noted otherwise.

## 5. Conclusions

Overall, in this study we demonstrated that the drug combination axitinib, erlotinib, and dasatinib displayed a potent and synergistic activity in pre-clinical models of colorectal cancer. We observed that the differences in cell line characteristics and expression profiles could be overcome by a combination of these drugs, leading to a comparable efficacy. We suggest a further evaluation of this drug combination in other preclinical models, to investigate whether the identified combination therapy could also be active against metastases, prevent metastasis formation, and whether it could be used to prevent drug-induced resistance.

## Figures and Tables

**Table 1:** CRC cell line characteristics.

Cell Line	Cancer Type	Origin	Mutations/Deregulations	Ref
SW620	Adenocarcinoma	Metastatic	APC, KRAS, TP53	[29,30,31]
HT29	Adenocarcinoma	Primary	APC, BRAF, PIK3CA, TP53	[29,32]
DLD-1	Adenocarcinoma	Metastatic	APC, KRAS, PIK3CA, TP53	[29,30,33]

## Supplementary Materials

The following are available online at https://www.mdpi.com/2072-6694/11/12/1878/s1. Figure S1: Combinations of axitinib, erlotinib, and dasatinib synergistically inhibit colorectal cancer cell metabolic activity in a dose-dependent manner. Figure S2: Variation in dose ratios of axitinib, erlotinib, and dasatinib combinations results in similar inhibition of colorectal cancer cell metabolic activity. Figure S3: Differential gene expression analysis of HT29 and SW620. Figure S4: Hematoxylin and eosin staining of SW620 and HT29 CAM tumors treated with axitinib, erlotinib, and dasatinib drug combination. Table S1. Comparison of the metabolic activity of SDC axitinib, erlotinib, and dasatinib on CRC cells, PBMCs, and HDFa. Table S2: Overview of methods used to grow tumors on the chicken chorioallantoic membrane (CAM) model.
