# Erratum: Berndsen, R.H., et al. Colorectal Cancer Growth Retardation through Induction of Apoptosis, Using an Optimized Synergistic Cocktail of Axitinib, Erlotinib, and Dasatinib, *Cancers* 2019, 11, 1878

**DOI:** 10.3390/cancers12051079

**Published:** 2020-04-26

**Authors:** Robert H. Berndsen, Nathalie Swier, Judy R. van Beijnum, Patrycja Nowak-Sliwinska

**Affiliations:** 1 Molecular Pharmacology Group, Institute of Pharmaceutical Sciences of Western Switzerland, University of Geneva, Rue Michel-Servet 1, 1211 Geneva, Switzerland; bobberndsen@hotmail.com; 2 Angiogenesis Laboratory, Department of Medical Oncology, Cancer Center Amsterdam, Amsterdam UMC-Location VUmc, VU University Amsterdam, De Boelelaan 1117, 1081 HV Amsterdam, The Netherlands; nathalieswier@hotmail.com (N.S.); judy.vanbeijnum@gmail.com (J.R.v.B.); 3 Translational Research Center in Oncohaematology, Rue Michel-Servet 1, 1211 Geneva, Switzerland

The authors wish to make the following corrections to this paper [1]:

The authors apologize for not giving sufficient credit to the involvement of Prof. C.R. Jimenez and her research group in this paper. Therefore, the following section is corrected: 

The “Acknowledgements” statement should be changed to:

**Acknowledgements:** The authors acknowledge Marloes Zoetemelk for the preparation of RNAseq material and Tse Wong for the expert technical support. The authors thank the iGE3 Genomics Platform (UNIGE) for preforming RNAseq experiments. The authors acknowledge Connie R. Jimenez and her team (Department of Medical Oncology, Cancer Center Amsterdam, Amsterdam UMC, Vrije Universiteit Amsterdam, Amsterdam, The Netherlands and OncoProteomics Laboratory, Cancer Center Amsterdam, Amsterdam UMC, Vrije Universiteit Amsterdam, Amsterdam, The Netherlands) for generating the phosphoproteomics data included in Figure 3.

The authors would like to apologize for any inconvenience caused to the readers by these changes.
