# Supplementary material for: Colorectal Cancer Growth Retardation through Induction of Apoptosis, Using an Optimized Synergistic Cocktail of Axitinib, Erlotinib, and Dasatinib
Source: Cancers (Basel). 2019 Nov 27;11(12):1878. doi: 10.3390/cancers11121878 (PMC6966484; doi:10.3390/cancers11121878)
Supplement: Supplementary file 1 [file cancers-11-01878-s001.pdf]

Article

Colorectal Cancer Growth Retardation through Induction of Apoptosis, Using an Optimized Synergistic Cocktail of Axitinib, Erlotinib, and Dasatinib

Robert H. Berndsen [1,2], Nathalie Swier [2], Judy R. van Beijnum [2] and Patrycja Nowak-Sliwinska [1,3,*]

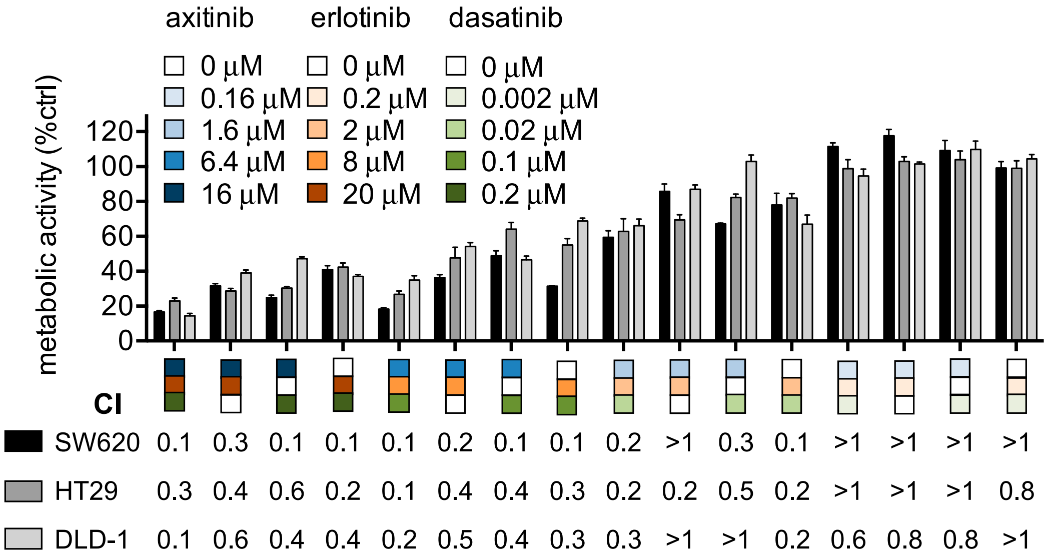

Figure S1. Combinations of axitinib, erlotinib and dasatinib synergistically inhibit colorectal cancer cell metabolic activity in a dose dependent manner. Metabolic activity of 2- and 3-drug combinations at multiple dose levels in CRC (SW620, HT29, DLD-1) cell lines. Color coded drug doses used include axitinib 0.16, 1.6, 6.4 and 16 µM, erlotinib 0.2, 2, 8 and 20 µM and dasatinib 0.002, 0.02, 0.1 and 0.2 µM and indicate dose reductions based on drug doses used on our previous study [1]. CI indicates Combination Index that was assessed with Compusyn software using the Chou-Talalay method [2]. CI values are showed below the graph. CI < 0.9 indicates synergism, CI = 0.9 indicates an additive effect and CI > 1 indicates antagonism. Metabolic activity was assessed after 72h of drug administration by the CellTiter-Glo® luminescence assay and represented as a percentage of control. Cells treated with 0.1% DMSO were used as control. Error bars indicate SEM.

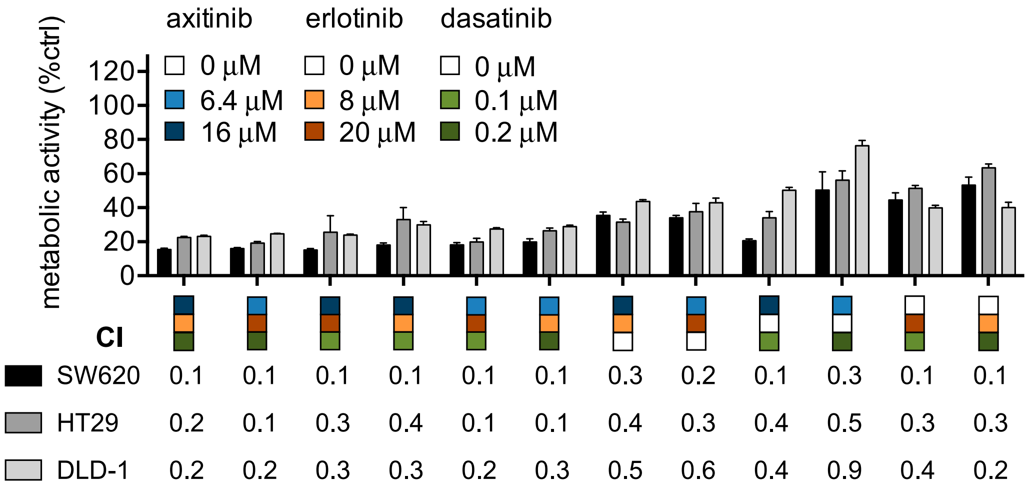

Figure S2. Variation in dose ratios of axitinib, erlotinib and dasatinib combinations results in similar inhibition of colorectal cancer cell metabolic activity. Metabolic activity of varying dose ratios of 2- and 3-drug in CRC (SW620, HT29, DLD-1) cell lines. Color coded drug doses include axitinib 6.4 and 16 µM, erlotinib 8 and 20 µM and dasatinib 0.1 and 0.2 µM and are based on our previous study[1]. CI indicates Combination Index that was assessed with Compusyn software using the Chou-Talalay method [2]. CI values are showed below the graph. CI < 0.9 indicates synergism, CI = 0.9 indicates an additive effect and CI > 1 indicates antagonism. Metabolic activity was assessed after 72 h of drug administration by the CellTiter-Glo® luminescence assay and represented as a percentage of control. Cells treated with 0.1% DMSO were used as a control. Error bars indicate SEM.

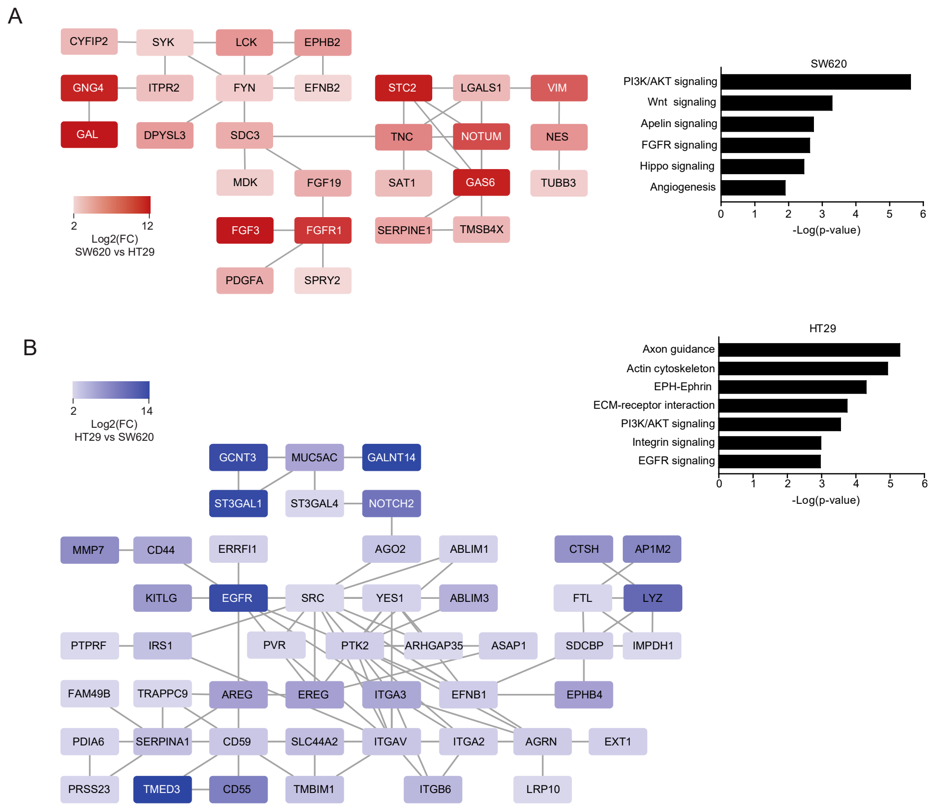

Figure S3. Differential gene expression analysis of HT29 and SW620. RNAseq data were analysed using DESeq2 in R for differentially expressed genes in SW620 (**A**) and HT29 (**B**). Gene lists were entered in String (String-db.org) and analysed for protein-protein interaction networks (confidence levels 0.7 and 0.9 for SW620 and HT29, respectively). Unconnected nodes were left out of the graphical representation. Nodes are color coded according to expression difference. Furthermore, gene lists

were entered in Enrichr (http://amp.pharm.mssm.edu/Enrichr/) for enrichment analysis on ontologies and pathways. Significant and relevant (>5 genes per group) features are represented in the bar graphs ordered by *p*-value.

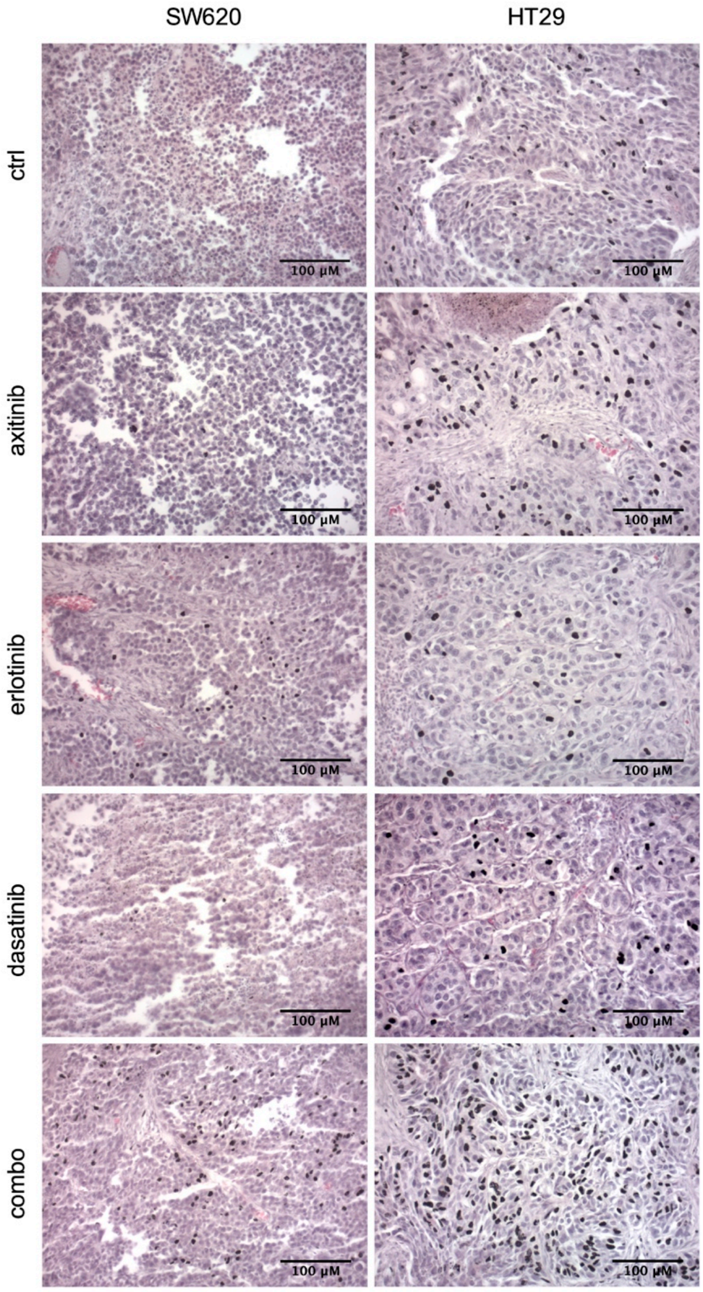

Figure S4. Hematoxylin and eosin staining of SW620 and HT29 CAM tumors treated with axitinib, erlotinib and dasatinib drug combination. Representative images of H&E stained tumor sections. Scale bars indicate 100 µm.

Table S1. Comparison of the metabolic activity of SDC axitinib, erlotinib and dasatinib on CRC cells, PBMCs and HDFa.

	**SW620**	**HT29**	**DLD-1**	**PBMC** [1]	**HDFa** [2]
SDC	15.2 ± 0.7	26.4 ± 1.8	13.8 ± 1.7	43.0 ± 2.2	48.0 ± 3.6

Metabolic activity was assessed after 72 h of drug administration by the CellTiter-Glo luminescence assay and represented as a percentage of control.

Table S2. Overview of methods used to grow tumors on the chicken chorioallantoic membrane (CAM) model.

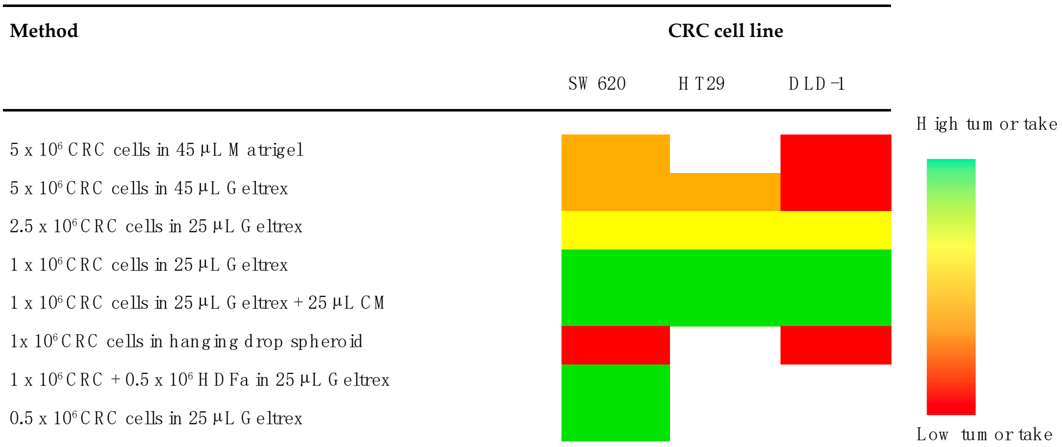

Green areas indicate the best tested method in regard to tumor take, survival of embryo's and tumor size. Red area's indicate the opposite. White spaces indicate that the method was not evaluated.

References

1. Weiss, A, Berndsen, R.H.; Ding, X; Ho, C.-M.; Dyson, P.J.; van den Bergh, H.; Griffioen, A.W.; Nowak-Sliwinska, P. A Streamlined Search Technology for Identification of Synergistic Drug Combinations. *Sci. Rep*. **2015**, *5*, 14508.
2. Chou TC. Drug Combination Studies and their Synergy Quantification Using the Chou-Talalay Method. *Cancer Res*. **2010**, *70*, 440–446.

© 2019 by the authors. Submitted for possible open access publication under the terms and conditions of the Creative Commons Attribution (CC BY) license (http://creativecommons.org/licenses/by/4.0/).
